# Association of Preoperative Shoulder Osteoarthritis Severity Score With Change in American Shoulder and Elbow Surgeons Score at 2 Years After Rotator Cuff Repair

**DOI:** 10.1177/23259671241257825

**Published:** 2024-07-31

**Authors:** Hannah M. Chi, Michael R. Davies, Chaiyanun Vijittrakarnrung, Daria Motamedi, C. Benjamin Ma, Brian T. Feeley, Drew A. Lansdown

**Affiliations:** * School of Medicine, University of California San Francisco, San Francisco, California, USA; † Department of Orthopedic Surgery, University of California San Francisco, San Francisco, California, USA; ‡ Department of Radiology and Biomedical Imaging, University of California San Francisco, San Francisco, California, USA; Investigation performed at the Department of Orthopaedic Surgery, University of California, San Francisco, San Francisco, California, USA

**Keywords:** SOAS score, ASES score, rotator cuff, shoulder arthritis

## Abstract

**Background:**

The impact of early glenohumeral osteoarthritis (GHOA) on clinical outcomes after rotator cuff repair (RCR) remains unclear. The magnetic resonance imaging (MRI)–based Shoulder Osteoarthritis Severity (SOAS) score is a comprehensive approach to quantifying glenohumeral degeneration.

**Purpose:**

To investigate the association between SOAS scores and changes in American Shoulder and Elbow Surgeons (ASES) scores in patients who underwent RCR.

**Study Design:**

Cohort study; Level of evidence, 3.

**Methods:**

Two reviewers independently analyzed the preoperative MRI scans of 116 shoulders and assigned SOAS scores. Spearman correlation was used to calculate the association of mean SOAS scores with patient demographic characteristics and change in ASES scores over the 2-year follow-up period (ΔASES). Multivariate regression analysis was performed between the independent variables of patient age, sex, body mass index, and significant SOAS score components as determined by univariate analysis, with the dependent variable being ΔASES. Significance was defined as *P* < .05 for univariate analysis and *P* < .0125 after application of the Bonferroni correction for multivariate analysis.

**Results:**

The mean ASES scores were 55.8 ± 18.6 preoperatively and 92.1 ± 12.1 at 2 years postoperatively. The mean preoperative SOAS score was 15.2 ± 7.1. On univariate analysis, the total SOAS score was positively correlated with patient age (*r*_S_ = 0.41; *P* < .001), whereas ΔASES was negatively correlated with patient age (*r*_S_ = −0.27; *P* = .0032). Increasing SOAS subscores for supraspinatus/infraspinatus tear size (*r*_S_ = −0.28; *P* = .024), tendon retraction (*r*_S_ = −0.23; *P* = .015), muscle atrophy (*r*_S_ = −0.20; *P* = .034), paralabral ganglia (*r*_S_ = −0.23; *P* = .015), and cartilage degeneration (*r*_S_ = −0.21; *P* = .024) were negatively correlated with ΔASES. A negative correlation was found between increasing total SOAS score and ΔASES (*r*_S_ = −0.22; *P* = .016). On multivariate analysis, increasing supraspinatus/infraspinatus tear size was significantly and negatively correlated with ΔASES (β = −3.3; *P* = .010).

**Conclusion:**

Increasing the total SOAS score was predictive of less improvement in ASES scores at 2 years postoperatively. On univariate analysis, SOAS subscores with the strongest negative correlations with ΔASES scores included tear size, muscle atrophy, tendon retraction, paralabral ganglia, and cartilage wear. On multivariate analysis, only tear size was significantly associated with a lower change in the ASES score.

Rotator cuff disease is the most common cause of shoulder pain and disability among adult patients, with the risk of tear increasing with age.^15,34^ The rate of rotator cuff repairs (RCRs) performed in the United States has increased as the population ages alongside advancements in arthroscopic surgical technique and diagnostic imaging.^18,33^ As more patients undergo RCR, studies have sought to identify factors associated with poor outcomes after surgery. It has been shown that worse patient-reported outcomes after RCR are associated with older age,^15,31^ increased body mass index (BMI),^10,35^ female sex,^1,10^ smoking,^2,3^ increased tear size,^4^ fatty infiltration,^11^ and any concomitant biceps tendon or acromial joint procedures.^14^

Glenohumeral osteoarthritis (GHOA) may coexist with rotator cuff tears in as many as 28% of patients, with a greater prevalence of OA in older patients and patients with larger tear sizes.^23,27^ However, the effects of GHOA on RCR postoperative outcomes have been controversial. Although some studies have shown moderate association between GHOA and rates of rotator cuff retear,^21^ others have found no significant differences between patients with or without GHOA in rates of retear, range of motion, and patient-reported outcomes.^19,28^ Kim et al^21^ found that although there were no differences in postoperative outcomes between patients with and without GHOA and small to medium rotator cuff tears, GHOA was associated with significantly decreased range of motion and worse patient-reported outcome scores in patients with large to massive rotator cuff tears.

GHOA is evaluated radiographically in most studies by classifications such as the Samilson and Prieto, Walch, Guyette, and Kellgren and Lawrence systems. These classification systems are widely used due to their excellent interobserver and intraobserver reliabilities. However, these radiographic systems characterize GHOA by hallmarks of more advanced disease such as joint space narrowing, osteophyte size, humeral head subluxation, and sclerosis; thus, they pose a challenge for the identification of early arthritis.^29^ Magnetic resonance imaging (MRI)–based scoring systems such as the Shoulder Osteoarthritis Severity (SOAS) score therefore represent potential alternatives in identifying and characterizing changes to soft tissue and inflammation associated with GHOA. The SOAS score ranges from 0 to 100, with 100 representing more severe pathology; there are 20 subscores of individual structures measuring severity of arthritic degeneration in 6 areas: rotator cuff, labral-bicipital complex, cartilage, osseous findings, joint capsule, and acromion.^20^ This semiquantitative whole-joint system has been shown to correlate strongly with established radiographic scoring systems and have excellent intra- and interobserver agreement.^20^ Recently, increasing SOAS scores were found to predict lower postoperative PROMIS-UE (Patient-Reported Outcomes Measurement Information System–Upper Extremity) scores in patients undergoing RCR.^5^

The purpose of this study was to use the American Shoulder and Elbow Surgeons (ASES) score to investigate the association between the degree of preoperative GHOA assessed by SOAS scores and changes in patient-reported outcomes among patients who underwent RCR. Because RCR is a joint-preserving procedure, we hypothesized that patients with more severe joint degeneration and higher SOAS scores before RCR would experience a smaller increase in ASES scores after surgery compared with patients who had lower preoperative SOAS scores.

## Methods

### Study Design and Participants

This was a retrospective cohort study from a prospectively collected database of patients who underwent arthroscopic RCR between 2018 and 2020. All patients were evaluated using the ASES score preoperatively and at 3 months, 1 year, and 2 years postoperatively. We included patients who underwent arthroscopic RCR with a preoperative MRI scan and at least 24 months of follow-up (n = 124). Patients with an MRI scan that was insufficient to calculate the SOAS score were excluded (n = 7), leading to a total of 116 shoulders from 116 patients that were included. This study was deemed exempt from institutional review board approval.

### Study Variables

Demographic characteristics of study participants (age, sex, and body mass index [BMI]) were recorded as well as preoperative ASES scores and ASES scores at 2 years postoperatively. MRI scans were scored by 2 independent reviewers (D.M., M.R.D.) using the SOAS criteria as described by Jungmann et al.^20^ The structures of the rotator cuff, labral-bicipital complex, cartilage, joint capsule, acromion, and osseous findings were individually scored to make up the overall total SOAS score (range, 0-100; higher scores indicate more severe degenerative changes). The interobserver reliability between the reviewers was calculated for total SOAS score as well as for each subscore.

### Statistical Analysis

Descriptive statistics including mean and standard deviation were calculated. Interobserver reliability of SOAS scoring was calculated using the intraclass correlation coefficient (ICC). The Spearman correlation was used to evaluate the association of mean SOAS scores (total score as well as each constituent subscore) with patient characteristics and change in ASES scores over the 2-year follow-up period (ΔASES scores). Multivariate regression analysis was performed between the independent variables of patient age, sex, BMI, and significant SOAS score components as determined by univariate analysis, with the dependent variable being ΔASES scores.

All statistical analysis was performed with Stata (Version 16.1; StataCorp). Significance was defined as *P* < .05 for univariate analysis and *P* < .0125 for multivariate analysis (m = 4) using the Bonferroni correction.

## Results

The study included 72 men and 44 women, with an overall mean age of 60.2 ± 10.3 years. The mean BMI of study patients was 27.3 ± 4.8 kg/m^2^. The mean preoperative ASES score was 55.8 ± 18.6, whereas scores at 2 years postoperatively were 92.1 ± 12.1 (Table 1). All patients had at least 24 months of follow-up. The mean preoperative SOAS score was 15.2 ± 7.1 (Table 2). The ICC for total SOAS score was 0.87 (Table 2).

**Table 1:** Characteristics of the Study Patients (N = 116)^*a*^

Characteristic	Value
Age, y	60.2 ± 10.3 (36-79)
Sex, n	
Male	72
Female	44
Body mass index, kg/m^2^	27.3 ± 4.8 (18.8-39.6)
ASES score	
Preoperative	55.8 ± 18.6 (16.7-93.3)
2-year follow-up	92.1 ± 12.1 (31.7-100)

a Data are presented as mean ± SD (range) unless otherwise indicated. ASES, American Shoulder and Elbow Surgeons.

**Table 2:** Total SOAS Scores, Subscores, and ICC Values^*a*^

SOAS Score Parameter	Score Range	Score, Mean ± SD	ICC^*b*^
Total SOAS score	0-100	15.23 ± 7.14	0.87
Rotator cuff		7.44 ± 4.37	
Supraspinatus/infraspinatus tear size	0-6	2.75 ± 1.38	0.84
Subscapularis tear size	0-5	1.83 ± 1.02	0.89
Retraction	0-3	0.94 ± 1.0	0.89
Fatty infiltration	0-4	1.48 ± 1.43	0.99
Atrophy	0-3	0.43 ± 0.89	0.97
Labral-bicipital complex		1.97 ± 1.29	
Labrum	0-2	0.91 ± 0.69	0.83
Long head of biceps	0-3	0.98 ± 0.85	0.98
Paralabral ganglia	0-3	0.026 ± 0.17	0.16
Glenohumeral ligaments	0-2	0.056 ± 0.22	0.0
Cartilage		1.22 ± 1.76	
Glenohumeral articular cartilage	0-6	1.22 ± 1.76	0.94
Osseous findings		1.60 ± 1.42	
Bone marrow edema	0-3	0.98 ± 0.96	0.68
Subchondral cysts	0-3	0.39 ± 0.70	0.92
Osteophytes	0-3	0.21 ± 0.41	0.37
Bone deformity	0-3	0.017 ± 0.11	0.0
Joint capsule		1.09 ± 1.03	
Synovitis	0-2	0.60 ± 0.63	0.79
Joint effusion	0-3	0.46 ± 0.57	0.54
Loose bodies	0-2	0.026 ± 0.15	0.24
Acromion		1.93 ± 0.91	
Subacromial bursitis	0-3	0.53 ± 0.42	0.085
Acromioclavicular joint degeneration	0-3	1.40 ± 0.75	0.89
Acromion deformity	0-2	0 ± 0	—

a ICC, intraclass correlation coefficient; SOAS, Shoulder Osteoarthritis Severity.

b *P* < .05 for all ICC values.

Correlation analysis indicated that total SOAS score was positively correlated with patient age (*r*_S_ = 0.41; *P* < .001), whereas ΔASES was negatively correlated with patient age (*r*_S_ = −0.27; *P* = .0032). A negative correlation was found between increasing preoperative SOAS score and ΔASES (*r*_S_ = −0.22; *P* = .015) (Table 3). Increasing supraspinatus/infraspinatus tear size, tendon retraction, muscle atrophy, paralabral ganglia, and cartilage degeneration were all negatively correlated with ΔASES (Table 3).

**Table 3:** Summary of Correlations^*a*^

Comparison	*r*_S_	*P*
Patient characteristics		
Age vs total SOAS	0.41	**<.001**
Age vs ΔASES	−0.27	**.0032**
Sex vs total SOAS	−0.059	.53
Sex vs ΔASES	0.0082	.93
BMI vs total SOAS	0.13	.18
BMI vs ΔASES	0.094	.32
Total SOAS score vs ΔASES	−0.22	**.016**
Rotator cuff		
Supraspinatus/infraspinatus tear size vs ΔASES	−0.28	**.0024**
Subscapularis tear size vs ΔASES	−0.021	.82
Retraction vs ΔASES	−0.23	**.015**
Fatty infiltration vs ΔASES	−0.084	.37
Atrophy vs ΔASES	−0.20	**.034**
Labral-bicipital complex		
Labrum vs ΔASES	−0.17	.075
Long head of the biceps vs ΔASES	−0.17	.068
Paralabral ganglia vs ΔASES	−0.23	**.015**
Glenohumeral ligaments vs ΔASES	0.085	.37
Cartilage		
Cartilage vs ΔASES	−0.21	**.024**
Osseous findings		
Bone marrow edema vs ΔASES	−0.078	.41
Joint capsule		
Joint effusion vs ΔASES	0.015	.87
Loose bodies vs ΔASES	−0.030	.75
Acromion		
Subacromial bursitis vs ΔASES	−0.028	.76
Acromioclavicular joint degeneration vs ΔASES	−0.0064	.95

a Boldface *P* values indicate statistical significance (*P* < .05). ΔASES, American Shoulder Elbow Surgeons score 2-year follow-up period; BMI, body mass index; SOAS, Shoulder Osteoarthritis Severity score.

In the multivariate linear regression analysis of significant SOAS variables with patient characteristics to predict ΔASES, only increasing supraspinatus/infraspinatus tear size was found to be significantly negatively correlated with ΔASES (β = −3.3; *P* = .010) (Table 4).

**Table 4:** Multivariate Regression of SOAS Variables to Predict ΔASES^*a*^

	β	SE	*P*
Age	−0.31	0.18	.088
Sex^*b*^	−0.83	3.54	.82
BMI	0.42	0.36	.25
Total SOAS	−0.40	0.26	.13
Age	−0.36	0.17	.031
Sex^*b*^	−2.23	3.52	.53
BMI	0.45	0.35	.21
Supraspinatus/infraspinatus tear size	−3.30	1.25	**.010**
Age	−0.35	0.17	.042
Sex^*b*^	−1.99	3.54	.58
BMI	0.50	0.36	.17
Tendon retraction	−4.08	1.78	.024
Age	−0.31	0.18	.077
Sex^*b*^	−0.63	3.52	.86
BMI	0.43	0.36	.24
Atrophy	−3.99	2.01	.050
Age	−0.39	0.17	.021
Sex^*b*^	−0.27	3.51	.94
BMI	0.20	0.36	.59
Paralabral ganglia	−21.52	10.11	.036
Age	−0.39	0.18	.033
Sex^*b*^	−0.47	3.59	.90
BMI	0.33	0.36	.36
Cartilage	−0.60	1.05	.57

a Boldface *P* value indicates statistical significance (*P* < .0125 after Bonferroni correction for multiple comparisons). ΔASES, American Shoulder Elbow Surgeons score 2-year follow-up period; BMI, body mass index; SOAS, Shoulder Osteoarthritis Severity score.

b Female = 0; male = 1.

## Discussion

The results of this study in patients who underwent RCR indicated that higher preoperative SOAS scores were negatively correlated with change in ASES scores at 2 years after surgery. The SOAS subscores with the strongest negative correlations with ΔASES in univariate analysis were tear size, muscle atrophy, tendon retraction, paralabral ganglia, and cartilage wear. In a multivariate analysis with patient demographic characteristics, only supraspinatus/infraspinatus tear size maintained a significant negative correlation with change in ASES scores. These findings support the hypothesis that more severe preoperative degeneration of the shoulder joint is associated with lower patient-reported outcome scores after RCR.

Many other studies have demonstrated that the quality of muscle is an integral factor in predicting outcomes after RCR.^12,13,24^ We found that atrophy of the rotator cuff as well as degree of tendon retraction and tear size were both negatively associated with lower ΔASES after RCR. However, preoperative fatty infiltration on MRI scan was not negatively correlated with ΔASES on multivariate regression in this particular cohort. Fatty infiltration is a degenerative process that occurs in conjunction with other chronic changes of the rotator cuff after tear.^12,25^ In this study, the mean score for fatty infiltration was 1.48, indicating a low overall level of fatty infiltration in this cohort of patients. It is possible, therefore, that factors such as tear size, retraction, and muscle atrophy may be poor prognostic signs for RCR that present earlier in patients compared with fatty infiltration and may be more relevant in patients who are clearly indicated for a joint-preserving procedure such as RCR.

Interestingly, degeneration of the cartilage demonstrated significant negative correlation with change in ASES scores after RCR. Tears of the rotator cuff can lead to destabilization of the glenohumeral joint and subsequent degeneration of the cartilage and cuff tear arthropathy, especially in larger and chronic cuff tears.^8,17,26^ However, the effects of cartilage degeneration on patient-reported outcomes have been conflicting. In a prospective study with 33 patients, Klinger et al^22^ found that preoperative arthrosis before arthroscopic debridement of rotator cuff tears was prognostic of poor outcomes. A previous study with 45 patients found no significant differences in outcomes between patients who underwent RCR with and without cartilage degeneration.^20^ Notably, these studies used arthroscopic and radiographic methods to classify cartilage degeneration. Use of MRI to evaluate osteoarthritis via the SOAS classification may offer an improved method for assessing degenerative changes of cartilage as well as other bony and soft tissue changes that commonly occur in osteoarthritis compared with radiography and arthroscopy and may help guide clinical decisions and postoperative expectations after RCR in patients with damaged cartilage.

Prior work has demonstrated that more severe preoperative GHOA as classified by the SOAS system is associated with functional improvement after total shoulder arthroplasty.^6^ In contrast, the current study found that worse preoperative GHOA as quantified by MRI was associated with poorer patient-reported outcomes after a joint-sparing procedure. Reverse total shoulder arthroscopy is indicated in patients with rotator cuff arthropathy as well as patients with pseudoparalysis.^7^ Arthroplasty is rarely a first-line treatment option given the higher risk of complications and increased costs relative to nonoperative management and joint-sparing procedures^9,30,32^; however, when to perform a joint-sparing procedure versus a joint replacement in a patient with mild to moderate GHOA remains under debate. Radiographic classifications such as the Hamada classification continue to help inform these clinical decisions^16^; however, it may be possible to leverage the subtler findings on MRI to identify which patients would be better served with a joint replacement versus a joint-sparing procedure.

Although the SOAS score considers the different joint structures and periarticular structures, it is worth noting that rotator cuff characteristics make up one-third of the total score, and the rotator cuff subscores contributed largely to the overall SOAS scores of the patients included in this study with known rotator cuff pathology. Although cartilage degeneration was associated with decreased change in ASES score on univariate analysis, we found no correlation on multivariate analysis between change in ASES score and cartilage findings. Osseous findings and acromion structures similarly were not associated with change in ASES score on multivariate analysis. Therefore, although this study demonstrates a correlation between total SOAS score and change in ASES score 2 years after RCR, a direct correlation between change in ASES score and GHOA may be less clear.

The complete clinical picture including patient age, muscle quality, and joint degeneration should be considered when discussing treatment options, and use of MRI-based tools such as the SOAS score may prove useful in guiding these clinical decisions.

### Limitations

This study has several limitations. Although the interobserver reliability for total SOAS score (ICC = 0.87) demonstrated good agreement, the ICCs for several subscores demonstrated low reliability. It is likely that this was due to certain parameters of the SOAS score, such as bony deformity of the humerus or glenoid, being exceedingly rare findings in this cohort of patients. Additionally, the strength of the correlations between SOAS score components and ΔASES scores was most frequently in the weak to moderate range. This may reflect the fact that the overall magnitude of joint pathology in this cohort of patients indicated for a joint-sparing surgery was low; on the SOAS scale from 0 to 100, the mean score for patients was 15.2. The utility of the SOAS score in predicting outcomes for patients with more severe osteoarthritis is uncertain, and future studies should focus on patients with more significant degenerative changes for which indications for joint-sparing versus joint-replacing procedures are less clear. Finally, the application of the Bonferroni correction to the multivariate analysis increases the risk of a type 2 error, which may limit the conclusions drawn from that analysis.

## Conclusion

In this prospective cohort of patients undergoing RCR, increasing preoperative total SOAS score was predictive of less improvement in ASES scores at 2 years after surgery. On univariate analysis, SOAS subscores with the strongest negative correlations with ΔASES scores included tear size, muscle atrophy, tendon retraction, paralabral ganglia, and cartilage wear. Tear size alone was found to be significantly associated with a lower ΔASES in multivariate linear regression analysis.
